# The Construction and Government of Lunatic Asylums and Hospitals for the Insane

**Published:** 1847-07

**Authors:** 


### Art. X.

*The Construction and Government of Lunatic Asylums and Hospitals for the Insane.* By John Conolly, m.d., &c. With Plans.—*London,* 1847. 8vo, pp. 184.

Led by the association of subject, on laying down the book which formed the basis of our last article, we very naturally took up the present: both books treating of Insanity; and both the authors being physicians who are known to have been engaged in the treatment of this disease. But what a contrast between the books! Dr. Conolly's possesses every merit which Dr. Seymour's wants. It has, indeed, as many merits as the latter has defects, and we could hardly pay it a higher compliment. The subjects treated of by Dr. Conolly are just as clearly conceived and as logically arranged as Dr. Seymour's subjects are the reverse; and the style in which the information is conveyed is to the full as accurate, clear, and elegant, as Dr. Seymour's is inaccurate, obscure, and vulgar. In a word, if we have said that Dr. Seymour's book is creditable neither to its author nor to our literature, we say, with equal confidence, that Dr. Conolly's is in the highest degree creditable and honorable to both.

Although the contents of Dr. Conolly's little volume are strictly in accordance with its title, and treats mainly and most fully of everything relating to the Management and Government of Asylums, it also contains, incidentally but necessarily, a great deal on the general treatment of the insane. It must not therefore be imagined that it is exclusively interesting to those having immediate connexion with asylums. To all, indeed, who have such a connexion, whether lay or professional—whether as physicians, surgeons, chaplains, matrons, attendants; or as governors, magistrates, visitors, committeemen, projectors, or architects of asylums, —it must prove invaluable, as containing all that they require to know, and much of the greatest possible importance that they should know, and which they cannot find elsewhere. But to the medical profession generally, it presents not only a subject of great interest admirably handled, but it offers much valuable matter relating to the character of the insane and the mode of treating them, which every member should be acquainted with.

In a prefatory note the author tells us that "the greater part of the book is a reprint from Lectures published in the Lancet, in 1846, supplementary to a clinical course on the symptoms and treatment of the various forms of insanity." We should much more regret that Dr. Conolly has restricted the republication to these few lectures, did we not entertain a hope that he will give us the others on the general subject of insanity, on some future occasion, in an enlarged form. To him, of all men, the great and influential promulgator and promoter, if not the actual introducer, of the humane system of treating lunatics, which does such honour to our time, the profession looks for information on all that relates to the nature and cure of insanity. As members of that profession, we do so likewise; and we shall, therefore, choose to regard the present publication—invaluable as it is—only as a small instalment of the great sum of information which we would fain fix on our author as an imperative debt to his brethren, yet to be cancelled.

As it would be impossible to convey to our readers the manifold minute yet most important details respecting the construction and arrangement of asylums, without transcribing the greater portion of the chapters devoted to these, we must content ourselves with a strong recommendation to all interested in these subjects, to study the original. As we go over the different chapters we shall merely pick out, here and there, some of those passages which have a more general bearing on the treatment of insanity. These will speak for themselves as to the elegant and refined style of the work, and will give a general idea of the gentle and philanthropic spirit in which it is conceived. They will, however, convey but a faint impression of the depth and tenderness of the sympathies of the author towards the poor and beloved subjects of his care, with which the whole volume abounds. The book, indeed, presents us with a striking contrast in the manner in which the author speaks of his patients and their attendants on the one hand, and of the governing and ruling authorities of asylums, on the other. He uses the utmost freedom in commenting on the proceedings and shortcomings of governors, matrons, &c., but he lets slip no occasion of defending the claims and rights of his poor patients and their hard-worked attendants,—or rather, we should say, his feelings of affection and sympathy towards them, well out from his mind on all occasions unconsciously, and irresistibly.

The following extract is the conclusion of the author's obsevations respecting the best site for an asylum; it shows how his mind delghts to take the loftiest as well as the gentlest views of things.

"Patients of all classes derive advantage from the circumstances of situation; and if it is intended to receive patients of the educated classes into the house, it should unquestionably be situated amidst scenery calculated to give pleasure to such persons when of sane mind. Those whose faculties have never been cultivated derive little satisfaction from the loveliest aspects of nature, and experience little emotion amidst the grandest. The sun rises and sets, the stars shine and fall, the hills reflect all the variety of brightness and shadow, of wildness and of verdure, and yet are scarcely noticed with more than mere passing attention. But when education has called the higher faculties into life, impressions upon them, even from external nature, become powerful for good or ill, and in the case of a mind diseased, may act as remedies, or aggravate the malady. The celebrated Robert Hall attributed much of his unhappy state of mind, and even his temporary insanity, to a change of residence from a picturesque and interesting part of the country to a cheerless plain, of which the dulness, flatness, and invariable monotony saddened his heart. Cowper, whose writings indicate exquisite sympathy with the sights and sonnds of common rural retirement, and who, like Robert Hall, was occasionally afflicted with insanity, felt awe-struck and overwhelmed when visiting a friend whose house was situated among lofty hills covered with trees. There are few persons of any degree of education much of whose daily and habitual pleasure does not arise from the view of the objects around them; and the first desire of all who can quit the crowd and toil of business, is to be where they can enjoy 'a prospect,' or to surround their houses with shrubs and flowers. Even in the populous city, the pent-up artisan has a bird, to sing to him whilst he works, and a few flowers, which he cultivates with care. We must not neglect such instincts and capacities if we profess to cure diseased minds. Our practice can only securely rest on the consideration of everything, great or little, capable of affecting the mind beneficially or hurtfully." (pp. 9-10.)

The following extracts show the importance of minute things as calculated to affect the feelings of patients:

"Much ornament or decoration, external or internal, is useless, and rather offends irritable patients than gives any satisfaction to the more contented. In some of the Italian asylums, busts, pictures, and ornaments abound, and the walls are painted with figures representing various allegories or histories. These would appear more likely to rouse morbid associations than to do any good. I also disapprove of painting numbers and titles on the walls of airing-courts, by which the walls are disfigured, and the patients are reminded of their confinement as insane persons when walking out for relaxation, or led to consider themselves prisoners. The wards and bedrooms also should only be designated by simple numbers.

"When it is remembered that many patients are sent to an asylum whose senses are as perfect, and whose feelings are as acute, as those of sane people, and that from the moment they enter the outer gate everything becomes remedial with them, or the reverse, the reason will at once be seen why the external aspect of an asylum should be more cheerful than imposing, more resembling a well-built hospital than a place of seclusion or imprisonment. It should be surrounded by gardens or a farm. Even the part of the building to which the patient is first admitted is important. In the new asylum about to be built in the county of Middlesex, I feel sure that the magistrates will anxiously avoid an inconvenience which is very much felt in the present building, where the reception rooms open directly into large towers, in which a circular staircase is so guarded with iron palisades as to give the patients looking through them, in the three different stories, the appearance of persons shut up in tiers of iron cages. A very painful impression is often made by this prospect being suddenly presented to the new comer; and it is strongest in those in whom the hope of recovery renders the avoidance of all counteracting agencies of the most consequence. The reception-room should be a cheerful and neatly-furnished sitting-room, and so situated that the newly-admitted patients can proceed from it to whatever part of the asylum it is thought best they should be sent. Thus, violent and noisy patients would not be led shouting and struggling through tranquil wards; and timid and quiet patients would not have to pass along corridors containing patients whose unfamiliar looks agitate or affright them." (pp. 13-14.)

"All the locks in the house should be so made, and so kept in order, as to open and close with the least possible noise. The jingling of keys, the clang of the locks, and the violent opening or shutting of the doors of bedrooms and galleries, are generally considered of no consequence by the attendants; but they produce the most uncomfortable feelings in the patients. The gallery doors should have large handles, enabling those who pass through to shut the door quietly. The state of a whole ward may immediately be altered by persons hastily and noisily passing through. There is, however, no point so little attended to by the generality of attendants, servants, workpeople, officers, and even by the official visitors of all asylums. The physician's caution is generally despised, and his example disregarded. Doors are thrown suddenly open; attendants are loudly or fiercely called for; unexplained intimations are given of something being wrong somewhere; the ward is hastily passed through; the hindering patients are put aside; the door is violently shut; the next ward and the next are similarly invaded; and the whole of the inmates, attendants as well as patients, are left in a state of agitation, or even of alarm. I have often, in former times, discerned the impression of these impetuous visits so distinctly left as to make me inquire into its cause; and have had deep reason to lament that it should be so much forgotten that the manner, the voice, the words, the very step of those who pass through a house full of insane people ought to be calm and tranquillizing. Those most conversant with the insane will the most readily acquit me of exaggerating the importance of these matters." (pp. 29-30.)

*Cruel economy.* "There is a refinement of economy practised in some private asylums which grievously interferes with the comfort of educated patients, and which consists of allowing a long interval to elapse between the departure of daylight and the introduction of lamps and candles. A great amount of suffering is entailed on the patients by this ingenious neglect. This is generally allied with an equal anxiety to save in the article of fuel; and servants are permitted to forget to mend the fires with entire impunity. The same principle creeps sometimes into public asylums; and to save the expenditure of a few shillings in a week, the patients are allowed to grope their way about the wards, and find their way to bed as they can, when the days are shortening, and when the patients are also cold and comfortless and irritable, in consequence of passing many hours in too low a temperature." (p. 37.)

*Importance of cleanliness.* "The provisions necessary for cleanliness are humble things to dwell upon, but they are the auxiliaries of health, and deserve the most vigilant attention of the physician, who ought to remember, when he detects a bad smell in passing any door in a gallery or passage, that some of the inmates of the asylum are exposed to that offensive air all the day long, and all the night, and that some of them never go out beyond the boundaries of the asylum grounds, so as to recruit, by change of air and scene, their constitutional power of resisting the influence of local malaria. And whoever pays much attention to the habits of human beings, must know how much the irritability of the mind is increased by habitual ill health, in whatever way produced: and it is with this irritability, on a large scale, that we have to deal, and which it is our business to remedy or prevent in asylums for the insane. . . . . . . . . . . There should be in every ward a lavatory or washing-room, containing half-a-dozen enameled-iron washing basins, sunk in a leaden table, and each basin supplied with a watercock, and having a moveable plug. There should also be one or two large round towels in each room. Among the late additions to the wards at Hanwell, none has been more productive of comfort to the patients than this, and the washing-rooms are the perfection of neatness. These rooms are open to the patients of quiet wards at all hours of the day; and the means of having a clean face and hands, and the refreshment of washing when fatigued, or after various occupations, is very much enjoyed. An additional means of personal cleanliness is also afforded by each patient having a warm bath once a week. Where these regulations prevail, the skin of insane patients loses its most repulsive and unwholesome aspect; general health is promoted, and with health, comfort, and cheerfulness; and the peculiar odour of the insane, so often described, does not permeate every ward, and is, indeed, seldom perceived. There are private asylums, on the old plan, which I could recognize as such, if taken blindfolded into them; and where this terrible and peculiar smell tells the visitor, as plainly as any impression on the sense can tell, that the rooms are not thoroughly clean, that the bed and body linen are not sufficiently often changed, and that washing and bathing are very little attended to." (pp. 38-40.)

The following extracts will be read with deep interest, as giving us a glimpse of the early scenes witnessed by Dr. Conolly at Hanwell, and the effect they had on his mind.

"It was in the female infirmary at Hanwell, exactly seven years ago, that I found, among other examples of the forgetfulness of what was due either to the sick or insane, a young woman lying in a crib, bound to the middle of it by a strap round the waist, to the sides of it by the hands, to the foot of it by the ankles, and to the head of it by the neck: she also had her hands in the hard leather terminations of canvas-sleeves; she could not turn, nor lie on her side, nor lift her hand to her face; and her appearance was miserable beyond the power of words to describe. How long she had been in this state it is not material to record. That she was almost always wet and dirty, it is scarcely necessary to say. But the principal point I wish to illustrate by mentioning this case is, that it was a feeble and sick woman who was thus treated. At that very time, her whole skin was covered with neglected scabies, and she was suffering all the torture of a large and deep-seated abscess of the breast. Let it be considered what must be the effect on the attendants of having customary recourse to the imposition of restraints, when such complicated suffering as this became comparatively disregarded by medical men, in consequence of the spectacle presented to them being, at each visit, not that of a sick person requiring aid, but of a dangerous lunatic cruelly fastened and bound. But this patient was neither dangerous to herself nor to others. The excuse alleged for this mode of treatment was, that she would eat the poultices employed, and which contained lead, and that she was very mischievous: that was all. However, she was liberated; no bad consequences ensued, and in a few weeks I saw the poor creature at the chapel, and even heard her play the organ, which she had been accustomed to do in the church of a village in Middlesex before her admission. This patient died very recently; having from the time of her liberation from restraints scarcely ever given any trouble to the attendants. Perhaps if I had never seen such a case, I should have been less earnest to adopt the system which I knew had been tried at Lincoln, and slower to try to manage the patients of this great asylum entirely without restraints. Many a case was yet to be managed in which every ingenious difficulty was created or encouraged to baffle this attempt; many anxieties were to be endured, many misapprehensions to be submitted to, and much suffered: but all is now passed; and I thank God, with deep and unfeigned humility, who has permitted this great experiment to proceed, for full seven years, without one accident calculated to discredit it; and with a general result on the asylum best known to those who knew the asylum before; and a general effect on all other asylums in almost every region of the globe, which can never be entirely lost." (pp. 47-8.)

"Those who now visit the asylum, and who witness no such scenes, may be excused for imagining that I am needlessly apprehensive of the use of restraints being revived, and may talk, with apparent reasonableness, of the occasional and moderate employment of such methods. Experience has taught me that their shocking abuse, if once more witnessed, would subvert all such reasonings. Their abuse, to an extent which now can scarcely be described without a suspicion of exaggeration, and in an asylum distinguished from its first opening by the comforts and advantages it extended to the pauper lunatic, shows that their use, to any extent, cannot be permitted without danger. Attendants trained in asylums where restraints are used, immediately apply them in private and recent cases, thus producing the worst consequences. Of this I meet with frequent and lamentable instances in private practice; where days, precious to the treatment of recent cases, are passed in all the misery and disadvantage of bonds, and dirt, and darkness, and unregarded fever, restlessness, and thirst." (pp. 46-7.)

We must take a few extracts from the pages devoted to the very important subject of *exercise in the open air*.

"Both with reference to health and recreation, as well as to employment, the airing-courts and grounds and gardens of an asylum are worthy of especial attention. A most gratifying improvement has taken place in these respects also, within the last few years, in many asylums. In the older institutions these had no place, or dismal yards and barren courts were alone to be seen. No external influence could have been devised more powerful to depress the mind, and sink it into inactivity, than monotonous gravel courts, surrounded by walls from ten to fifteen feet high; without a tree; without a shrub; without a blade of grass; without shade in the heat of summer, or shelter from the rains of winter—the only luxury being a bench fastened to the wall, with large iron rings suspended over it, so that even in the open air restraint might still be substituted for superintendence. In these respects, asylums were, until very lately, precisely like jails, in one of which a man, who had been long imprisoned for an act committed in a fit of insanity, used to say to me, when I visited him,—

'Sir, I have not seen a flower or a green leaf these seven years!'—a painful observation to the ears of those who can pass from such gloomy precincts to fields and pleasant footpaths, never more to be trodden by the miserable. Within equally melancholy boundaries might be seen, in most asylums, but a few years ago, every form of gloom and eccentricity which the absence of all external objects of interest could foster; and the patients walked up and down some chosen path beneath the hopeless walls, until the very ground was worn into hollows; or, debarred from the full exercise of the muscles of locomotion required by their excited brain and nerves, expended all their energy in exertions of voice, distressing to all within hearing. Even at present, such arrangements may yet be witnessed, and the consequent concentration of morbid excitement of a crowd of insane people, which ought rather to be allowed relief by action and expansion in liberal space." (pp. 49-50.)

"It is astonishing to find that one of the particulars of treatment in which the rich patient is sometimes more unfavorably situated than the poor, is that of having the opportunity of enjoying free and frequent exercise. The untrodden lawn, the dusty and desolate courts, the paved yards, the wretched sheds, the lonely outhouses, together with the closed doors and windows of a private asylum, often give to such places an external character which makes the visitor regard them with dread, and the passer-by speak of them in whispers. The idea of their entrance is connected with that of the fatal gate, which whoso entered left all hope behind. If a patient is visited in such a house, the unlocking of doors, and the threading of long passages, and ascent of gloomy stairs, with the close atmosphere of apartments filled with patients sitting by the walls, oppressed with indolence and monotony, are all features too familiar to those who knew such houses before later improvements penetrated into them. And it is only in a few of the best private asylums that we even now find cheerful sitting-rooms on the ground floor, opening on gardens, into which the patients may walk when they please; and the benefit of this improvement is yet too generally limited to the most rational of the patients, and not extended to the irritable and troublesome, who ought also to be able to go out of their sitting-rooms, although into gardens more secluded and secure. The extent of ground assigned to the patients, their subdivision during exercise, and, indeed, all the other arrangements in private asylums, vary so much in different establishments in these days of movement, that the remarks justly applicable to several which have adhered to the ancient ways, would be most unjust, as well as painful, to the proprietors of some, in which no sacrifice has been spared to secure for every patient all the advantages which a physician or a conscientious superintendent would wish them to enjoy." (pp. 58-9.

The following, out of many admirable remarks on the subject of *dress*, we recommend to all proprietors and superintendents of private and public asylums.

*Dress of Nurses.* "The number of female patients who go about bareheaded is always greatest where there is the most neglect. It is not natural to the woman to neglect the dress of her head, and if the faculty is impaired, care will often restore it. When convalescence is commencing, the patient generally becomes more cheerful if some assistance is given her as regards her dress for Sunday; and of this a neat, or even a pretty cap, is an important part. The men also, when recovering, often ask for newer clothing to go to chapel in. The attendants should set a good example. Slovenly attendants generally increase the number of dirty patients. The male attendants should not be permitted to wear loose holland frocks, which give them the appearance of butchers; and the female attendants should not be discouraged from observing the neatness and taste in dress which are natural to them. No mistake is greater than that of supposing that being dressed in unbecoming clothing, in stuff gowns and in mob-caps, is either a virtue in itself, or an incentive to virtue. Such sentiments form part of a gloomy and selfish system, including mortifications and degradations, especially unfavorable to goodness of any kind, and only gratifying to those who impose them. Such dresses are especially distasteful to English women of any class; they always discard these outward signs of poverty as soon as they are raised above the lowest condition of pauperism. A neglect of this really proper feeling is a frequent cause of discontent in asylums, and sometimes retards recovery." (pp. 61-2.)

*Dress of private patients.* "Many private asylums are open to the charge of great neglect as respects the dress of patients of the classes far above pauperism. Tattered and threadbare coats, very shabby hats, trousers not always free from an offensive smell; and equally slovenly dresses on the female side of the asylum, shoes out of repair, hair in curl-papers, make the unfortunate patients objects of pity or of ridicule. They feel themselves degraded, lose their self-respect, and with it the little self-control their malady has left them. It is very true, that in some cases the patients will not dress themselves properly, that they have an affection for old and ragged garments, and insist upon their being fit to go to court in, and are violently offended if better clothes are substituted for them; but such cases form only a small proportion in any asylum; and in many instances, habits of personal neatness may long be preserved, and in some restored, after being long lost. Even an apparent disregard for cleanliness sometimes proves to have been the result of previous neglect and ill-treatment. Patients are often brought to Hanwell who are reported to us as uncleanly, but who do not prove so when properly attended to. Others who have been pronounced uncleanly, and sent to wards appropriated to such patients, have recovered the power of keeping themselves neat, and avoiding any uncleanly habits, after some accident has caused them to be subjected to the superintendence, and benefited by the care, of the attendants in the infirmaries. Such cases are not very frequent, but they prevent despair in circumstances which appear hopeless.

"The rule should be, in private asylums, that each gentleman should be encouraged to dress according to his station, and to be at least as careful of his whole dress, his boots, his hat, his gloves, as he used to be when well. Ladies should not be allowed to forget that they are ladies; but should be required to dress appropriately, both in the morning and for dinner. Their friends are sometimes more in fault than they, and the patients are disfigured against their will; but it is disadvantageous to them to be thus permitted to fall into a negligence characteristic of advanced and incurable forms of disorder." (pp. 63-4.)

The following is the only extract our limited space will allow us to take from the admirable chapter on *diet* and *employment*.

"It is impossible to be too careful in directing that all the service of the table should be in accordance with the habits of the patients. The sense of banishment from home, and of confinement, and the consciousness of mental infirmity and dependence, are mitigated in the mind of many a silent, uncomplaining patient by these means. All the comforts added of late years, and with so much advantage, to the wards of county asylums—such as moveable tables and chairs, window-blinds, plants, musical instruments, bagatelle boards, books, and pictures, with free access to agreeable gardens—show that the majority of the insane of any class are inclined to respect the decent arrangements made for them; and it will generally be found that the more conformable the furniture of the rooms, the tables, &c., of the higher class of the insane is to their habits and rank, the less they will be disposed to destroy or derange it. During violent paroxysms of mania, they are of course regardless of everything; and everything about them should be plain, simple, and secure. But when convalescence begins it should be respected. Among the depressing recollections of the insane of the higher classes, when recovering from insanity, I know that none are more frequent, or felt to be more degrading, than those connected with any want of respect shown to them, or any disregard of decent customs as to their meals. Yet, without great attention, they will sometimes be found, when quite well enough to appreciate what is done, sitting down to a dinner of meat, vegetables, and pudding, all sent to them on one plate. Negligences of this kind produce fretfulness and discontent, and tend to retard convalescence. If attendants are allowed to practise this kind of negligence, they soon fall into habits of rudeness, and even of inhumanity, fancying that the patients do not observe their conduct, and that their feelings are of no consequence." (pp. 76-7.)

Chapters V and VI are devoted to the subject of *attendants,* their qualifications, duties, and treatment, and contain a vast deal of information of the greatest importance as well as novelty. Here, as in every other part of his book, the author's sympathies are strongly enlisted on the side of the weakest. We could willingly transfer the whole to our pages, but we must be content with a very few brief extracts.

*Selection and duties of attendants.* "If I may rely on my own observation, no subject connected with the management of the insane, either in asylums or in private practice, has received less adequate attention than the selection of proper attendants, their proper treatment, their just government, and their instruction in the various, and peculiar, and exhausting duties which necessarily devolve upon them. The important and delicate task of regulating the conduct of persons of unsound mind, of controlling excitement, restraining waywardness, or removing mental depression, is unavoidably confided to persons of limited education; but these are too frequently chosen with little regard to their disposition, temper, or intelligence: they are permitted to commence their duties with as little preparation as if their office was merely that of a servant, and are governed either with severity and injustice, or without the consideration and indulgence requisite to support their patience, and to encourage them to be considerate and indulgent to those on whom they attend, and who are wholly in their power." (pp. 83-4.)

"An attendant must be expected to devote his whole time and attention to the patients; to consider himself the friend and adviser of those who are separated from every other friend and acquaintance, and the protector of those whom disorder of the mind has incapacitated from taking care of themselves. The functions they are to perform are not merely those of servants; they are the only sane persons always with the insane, and their temper, their manners, their cheerfulness and activity, their neatness and order, or the want of these qualities, will exercise a continual influence on all who are committed to their charge. On very many occasions I have noticed the remarkable effects produced on a patient, and often on a whole ward, by a change of attendant. Dirty patients have become cleanly, idle patients have become active, and morose patients have become cheerful. Contrary changes may not unfrequently be observed also, when the change is from a good attendant to a bad one." (p. 110.)

*Importance of good treatment of attendants.* "Attendants who zealously endeavour to perform all their duties well should be treated with confidence, and allowed every reasonable indulgence. The natural pride they feel in the state of their ward, and in their influence over troublesome patients, should not be discouraged by frequent removals either of the patients or of themselves from one ward to another. Everything that affects them will be found to exercise a secondary influence on the patients; their health, their circumstances, their instruction, their mode of living in the asylum, their diet, the comfort of their sleeping-rooms, are all matters of much importance, generally attracting too little attention, and sometimes wholly overlooked in the arrangements of an asylum. Yet nothing can be more certain, than that attendants who lead an uncomfortable life are not likely to be in a state of mind to make patients comfortable. If it is forgotten that they have ordinary human feelings, or if they are treated as if presumed to be always dishonest and unworthy of trust, or are subjected to heartless or capricious refusals and mortifications when desirous of a little extra leave of absence, they cannot but lose all attachment to the officers, and all interest in the asylum. It is a mockery to tell attendants, who are not sure of their places for a day, that they are to devote themselves entirely to the patients, and to be themselves patterns of forbearance. Yet the want of devotedness on their parts is a want of that for which there is no substitute, and the want of forbearance in the attendants will lead to the worst consequences. So strictly connected is the proper government of an asylum with the welfare, and even with the safety of the patients. Unjust officers, or unjust rulers, make attendants indifferent or cruel, and indifferent or cruel attendants make the patients wretched." (pp. 113-14.)

The last chapter considers the Religious Services of an Asylum—Schools—Government—Treatment of Officers—Qualifications and duties of the Superintendent.

The following beautiful passage shows at once the author's opinions as to the good and evil of religious observances in relation to insanity.

"The institution of religious services in asylums has created new and peculiar duties for the officers; and although I can readily conceive the apprehension with which the medical superintendents of some asylums regard this subject, and know how unjust it generally would be to ascribe such apprehensions to indifference, I am quite satisfied, that with reasonable caution in the exercise of his peculiar duties, a chaplain may become a valuable officer in asylums for the insane. It is unfortunately true, that no cause of mania, melancholia, and imbecility is more common than a gloomy religion, which excludes the idea of God's mercy so carefully, and brings forward God's judgments so prominently, as to alarm, and depress, and enfeeble many enthusiastic and weak persons who are exposed to its doctrines. Among persons of education, and particularly among women, I believe that nearly one half of the cases of derangement of mind arise from this perversion of religion alone. Exciting meetings, enthusiastic exhortations, false reports of wild missions, foolish biographies of sickly and delirious children, incoherent tracts, and books of unfruitful controversy, constitute all the intellectual exercises of these sincere and misguided persons. All elegant literature, and almost all science is kept from them, as demoralizing or tending to unbelief. All cheerful avenues of thought are forbidden to them. A restless, meddling, dictatorial spirit, much opposed to real charity, assumes the guise and name of benevolence and religious zeal. By degrees, the mind—so ill-exercised, so ill-governed, so excited—becomes weakened, and then the mask falls to the ground. Spiritual and worldly pride, idle prophesyings, convictions of eternal wrath, fierce denunciations of neighbours, or parents, or children, or relatives, and too often despair and attempts at self-destruction, declare that madness has supervened. Knowing all this, by daily observation, I feel as strongly as any physician can do, the danger of misapplying religious attentions; but I still believe that many insane persons are capable of deriving much satisfaction from being permitted to attend the services of their church; and that a good and prudent clergyman may become a useful auxiliary to a physician, by correcting fanatical delusions, moderating spiritual conceit, vindicating God from the unjust views of his creatures, and reviving every hope that is permitted to the imperfect and the penitent." (pp. 123-24.)

With the following extracts relating to the duties and qualifications of the medical superintendent of an asylum, we must take our leave of this fascinating volume. Our readers may probably be able to find a local habitation and a name for such offices and such an officer.

*Duties and qualifications of a superintendent.* "It is to him that the whole house must at all times look for the principles by which everything done in it is to be regulated. His supposed or his known wishes should be present to the mind of every officer and every attendant in every variety of accident, and his character of mind and heart ever in their view. Indifference on his part must lead to negligence on the part of those who execute his commands; severity exhibited by him must lead to brutality on the part of the attendants. His steady discouragement of negligence, his known abhorrence of cruelty, and his real and deep sympathy with his patients, may be reflected from every humane heart in the asylum.

"His duty comprehends a wide and careful survey of everything that can favorably or unfavorably affect the health of the mind or the body. He has to regulate the habits, the character, the very life of his patients. He must be their physician, their director, and their friend. The whole house, every great and every trifling arrangement, the disposition of every officer and servant, should be in perpetual conformity to his views; so that one uniform idea may animate all to whom his orders are intrusted, and the result be one uniform plan. Nothing should be done without his sanction. The manners and language of all who are employed in the asylum should but reflect his; for everything done and everything said in an asylum is remedial or hurtful; and not an order should be given, or a word spoken, except in accordance with the spirit of the director of the whole establishment. By such a system alone can it ever be proved to what extent the cure or the improvement of the insane is practicable.

"That he should be a person naturally benevolent is indispensable; and it is extremely desirable that he should possess an almost inexhaustible patience. The qualities to which, of old, much importance was attached—a commanding stature, a stern manner, a fierce look, a loud voice—have become either unnecessary, or positive disqualifications. Threats or reproofs, seconded by these attributes, may terrify the patients, but they loosen the bonds of affection, and generate feelings which will burst forth into expression in the next paroxysm, or revengeful designs, which will wait their time. Even remonstrances, to be successful, must be calm and carefully timed, being, as they are, addressed to the afflicted rather than the faulty. If sickness lays open all the delusions of life, madness often shows all human weaknesses magnified, and they must be viewed with never-failing charity, at no time forgetful that the dispositions so exhibited are impaired and deformed by insanity." (pp. 140-1.)

*Results and rewards of good management.* "If the whole of the system which I have imperfectly endeavoured to sketch be steadily persevered in, —no anger—no severity—no revenge—no deception—no disregard ever shown to the insane,—the resident superintendent will no longer find himself living among the habitually furious, or the incurably gloomy, or the constantly discontented. Calmness will come; hope will revive; satisfaction will prevail. Some unmanageable tempers, some violent or sullen patients, there must always be; but much of the violence, much of the ill-humour, almost all the disposition to meditate mischievous or fatal revenge, or self-destruction, will disappear. Some of the worst habits that beset the poor lunatic will also be got the better of; cleanliness and decency will be maintained or restored; and despair itself will sometimes be found to give place to cheerfulness or secure tranquillity. I could walk through such an asylum as I have described, and point out illustrations of every word in every ward.

"Resolved, therefore, to make his asylum a place where everything is regulated with one humane view, and where humanity, if anywhere on earth, shall reign supreme, the resident medical director must be prepared to make a sacrifice of some of the ordinary comforts and conventionalities of life. His duties are peculiar, and apart from common occupations. His society, even, must chiefly consist of his patients; his ambition must solely rest on doing good to them; his happiness on promoting theirs.

"None but those who live among the insane can fully know the pleasures which arise from imparting trifling satisfactions to impaired minds; none else can appreciate the reward of seeing reason returning to a mind long deprived of it; none else can fully know the value of diffusing comfort, and all the blessings of orderly life, among those who would either perish without care, or each of whom would, if out of the asylum, be tormented or a tormentor. Constant intercourse and constant kindness can alone obtain their entire confidence; and this confidence is the very keystone of all successful management.

"Thus living, and thus occupied, the director will learn to love his people, with all their infirmities, which are their afflictions. The asylum is his world. The patients are his friends; humble, but not without even delicate consideration for others; wayward, but not malignant, except when cruelty exasperates them; capricious, but not ungrateful; distrustful, but to be won by candour and truth; disturbed and grievously afflicted, but not dead to some of the best and purest affections. He will almost regard his patients as his children; their cares and their joys will become his; and humanly speaking, his whole heart will be given to them." (pp. 143-4.)

---

### ART. XI.

*Die Krankheiten des Zwerchfells des Menschen.* Von C. W. MEHLISS, M.D., &c.—*Eisleben,* 1845.

*The Diseases of the Diaphragm in Man.* By C. W. MEHLISS, M.D., &c.—*Eisleben,* 1845. 8vo, pp. 212.

